# Food Selectivity in Children with Autism Spectrum Disorder: A Statistical Analysis in Southern Italy

**DOI:** 10.3390/children10091553

**Published:** 2023-09-14

**Authors:** Angela Alibrandi, Agata Zirilli, Federica Loschiavo, Maria Cristina Gangemi, Alessandro Sindoni, Graziella Tribulato, Roberto Lo Giudice, Fausto Famà

**Affiliations:** 1 Department of Economics, University of Messina, 98122 Messina, Italy; angela.alibrandi@unime.it (A.A.); azirilli@unime.it (A.Z.); 2 Don Mottola Medical Center, 89862 Drapia, Italy; loschiavofederica17@gmail.com; 3 Independent Researcher, 98100 Messina, Italy; mariacristinagangemi89@gmail.com; 4 New Hospital of Prato S. Stefano, Azienda USL Toscana Centro, 59100 Prato, Italy; alessandrosindoni@alice.it; 5 Department of Human Pathology in Adulthood and Childhood “G. Barresi”, University of Messina, 98122 Messina, Italy; graziella.tribulato@virgilio.it (G.T.); fausto.fama@unime.it (F.F.)

**Keywords:** autism, food selectivity, pediatric population, statistical analysis, non parametric combination test, permutation solution

## Abstract

This paper focuses on autism spectrum disorder (ASD) and food selectivity, both of which are prevalent in the pediatric population. In this context, the authors paid attention to food selectivity and its possible correlation with the atypicality of sensory processes, outlining the useful rehabilitation treatments to draw on. This research included the parents or caregivers of pediatric patients diagnosed with autism spectrum disorder and placed within a therapeutic clinic. The sample is composed of 111 children, males and females, aged between 2 and 10 years, and includes 60 children diagnosed with autism and 51 children with normotypical development, similar in characteristics but without the disorder. The standardized questionnaire, “Brief Autism Mealtime Behavior Inventory”, was developed to examine behavior during meals, especially in children with ASD. The “Brief Sensory Profile”, and the “Child Oral and Motor Proficiency Scale”, were also administered. The results obtained from the analysis lead to evidence of eating and food selectivity difficulty. Additionally, our study demonstrates that food selectivity can be caused by extreme sensory modulation and sensory problems related to the smell, texture, color, and temperature of food. In fact, the results obtained emphasize the correlation between food selectivity and the sensory domains of taste and smell. Furthermore, this research highlights a correlation between motor skills and eating skills, particularly regarding food selectivity, which is closely associated with atypical and disruptive behaviors during meals.

## 1. Introduction

This paper focused on one of the disorders that now disproportionately affect the pediatric population. In fact, data in the literature indicate that one in 80 children has a diagnosis of autism spectrum disorder (ASD), with a higher percentage in the male gender. Genetics play a key role in the etiology of the disorder, although environmental factors should not be overlooked. The first description of the autism spectrum disorder dates back to 1943, when the neuropsychiatrist Kanner described early childhood autism, outlining its main characteristics [1]. He identifies autism with a heterogeneous set of behaviors and symptoms, which can have a large number of different causes and presentations [2]. In the past, childhood autism was considered quite rare, with prevalence estimates of 1 case in 2000–2500 subjects [3]. Subsequently, with the most recent “International Classification of Diseases” (ICD-11) and “Diagnostic and Statistical Manual of Mental Disorders” (DSM-V), more defined diagnostic criteria were used, which significantly contributed to modifying prevalence data [4,5]. The definition of more refined and standardized diagnostic procedures, which make it possible to detect even the slightest disorders, has created greater awareness among health professionals and the population, which has resulted in an increase in the number of health services responsible for detecting cases [6]. The origin of autism spectrum disorder is multifactorial [5] and can be caused by neurobiological, neurophysiological, genetic, neurofunctional, or environmental causes. Besides the symptoms that characterize the autism spectrum disorder phenotype, atypical behaviors are usually associated with it, although they are not exclusive to it and can also be present in several other clinical conditions. During the first year of life, social interaction is impaired and a lack of eye contact predominates. In facial expressions, atypicalities that regulate social interaction emerge, such as the absence of a smile or the poverty of mimicry, the presence of a smile, or a contextual cry. In the first year of life, body posture anomalies are also frequent. Many parents report difficulties in holding their child in their arms, and this is due both to the fact that the latter does not like physical contact but also to the inability of the child to adapt their posture to the posture of those who support them with their arms [4]. This inability is defined as a tonic disorder of dialogue. Impairment enriches social interaction and communication after the first year of life, in preschool age, with behaviors and their increasingly explicit and characteristic manifestations. The child tends to isolate himself; when he is called, he does not respond. The child with autism spectrum disorder, both within the family and outside of it, shows an inability and lack of interest in establishing relationships, isolates himself, or adopts inappropriate behaviors with passive participation or disturbing behaviors [7,8].

### 1.1. Food Selectivity

Food selectivity is a common issue among children with autism spectrum disorder [1]. This term encompasses various situations and behaviors, including rejecting certain foods, aversion to specific flavors, colors, textures, or temperatures, and sticking to a diet limited to specific food categories. Food packaging and presentation can also play a role [9]. Difficulty transitioning from paste to solid foods may be an early sign of a symptom of autism, such as restricted and stereotyped interests, which can manifest as pervasive eating disorders. Food selectivity is not limited to food refusal and lack of variety, but also includes a restricted diet of fewer than 8–20 dishes, indicating a strong rigidity in food choices and a poor acceptance of new foods [1]. This behavior is often accompanied by dysfunctional mealtime behaviors such as crying, yelling, running away, aggressive behavior, spitting, throwing food, and chewing without swallowing. Such ritualistic and repetitive behaviors contribute to defining food selectivity [10].

In healthy children, mealtimes play a vital role in their daily routine and development, as it helps children explore food according to a sensory dimension, acquire self-awareness, and reach a level of autonomy [1]. The meal time is also essential for developing specific skills, including social interaction and learning through imitation. Atypical eating behaviors can affect family mealtime routines [11,12], and for families of children with autism spectrum disorder, mealtimes can be overly focused on preparation and the child’s sensory experiences, which limits opportunities for shared experiences. Therefore, the treatment of food selectivity is crucial for children with ASD, especially as they often have difficulties with motor skills, articulatory ability, and speech and language development [13]. The treatment is multidisciplinary and involves various specialists, such as the child neuropsychiatrist, psychologist, nutritionist, and speech therapist, with the parents or family playing a central role [14]. Since selectivity is often associated with sensory integration problems, oral desensitization plays a fundamental role in gradually introducing new foods to the child [1]. The process may be challenging, but the goal is to proceed through gradual steps to achieve lasting and long-term results.

### 1.2. Scientific Background

Various studies have consistently demonstrated a higher prevalence of food selectivity in children with autism compared to those with normotypical development or diagnosed with ADHD [15,16,17]. These findings are supported by empirical evidence and a widespread scientific consensus, indicating a strong association between food selectivity and autism spectrum disorders. Notably, researchers have identified a link between restricted diets and the presence of ritualistic and repetitive behaviors, suggesting that food selectivity may be an extension of the rigidity and inflexibility commonly observed in individuals with autism.

Numerous factors contribute to food selectivity, prompting researchers to conduct extensive studies exploring the underlying causes. One significant factor is sensory sensitivity, also known as “sensory defense” or “sensory over-responsiveness.” Ayres and Tickle were among the first to describe sensory defense in relation to touch, defining it as an exaggerated reaction to certain tactile experiences, often resulting in aversion or negative behavioral responses to stimuli that most people would find harmless [18]. Children exhibiting tactile defense often struggle with being touched or interacting with tactile materials. This early tactile sensitivity likely contributes to feeding difficulties, particularly challenges with different food consistencies, observed in children with autism spectrum disorder.

Ornitz and Ritvo’s research highlights the inability of autistic children to tolerate certain tactile materials, such as woolen blankets or clothes in contact with their skin. It also emphasizes the high prevalence of sensory processing disorders across the autism spectrum and different age groups [19]. Leekam et al., in a sample of 200 children with ASD, reported a significant presence of sensory abnormalities, affecting multiple domains, particularly touch, taste, and smell [20,21,22].

The utilization of the Brief Autism Mealtime Behavior Inventory (BAMBI) questionnaire in multiple studies has facilitated the evaluation of feeding behavior in individuals with ASD, aiding both researchers and parents in identifying eating difficulties and implementing appropriate interventions [23,24,25,26,27].

By expanding our understanding of these factors and their impact on food selectivity, it is possible to develop targeted strategies to enhance the quality of life for children with autism and provide support for their families.

### 1.3. The Aim of This Paper

This paper aims to provide an overview of eating disorders in children with autism spectrum disorder, confirming the presence of selectivity, behavioral rigidity, refusal of food, and disruptive behavior with obsessive patterns during meals, compared to healthy control. At the same time, through validated and standardized questionnaires, the aim of this paper was to understand and evaluate a possible correlation between food selectivity and sensory integration in relation to touch, taste, smell, movement, search for sensations, a hearing filter, all energy, and auditory and visual sensitivity. Moreover, to individuate significant predictors of food selectivity, testing the effect of age, gender and ASD.

## 2. Materials and Methods

This research project enrolled patients with ASD from various medical rehabilitation centers, specifically the Italian Association of Spastic Assistance (A.I.A.S.) in southern Italy. During the study, the participants were already engaged in a clinical-therapeutic program that incorporated individual rehabilitation plans. These plans included short, medium, and long-term goals, along with specific treatments such as neuropsychomotor therapy and speech therapy. Furthermore, parents and/or caregivers were also included in the study, which focused on investigating food selectivity in individuals with autism spectrum disorder. The survey was conducted in the period between July and December 2022.

### 2.1. Inclusion Criteria

The inclusion criteria of the study involved patients affected by this disorder with a full-blown diagnosis of autism, carried out in hospitals and at territorial and extraterritorial research diagnostic centers. The presence of genetic syndromes such as Rett syndrome, fragile X syndrome, and Down syndrome, as well as other disorders such as epilepsies, sensory disabilities, brain injuries, metabolic disorders, and patients who followed specific disorders following gluten and casein-free diets, were excluded. The patients enrolled in this paper have a diagnosis of autism spectrum disorder. They were given an ADOS, “Autism Diagnostic Observation Schedule”, a widely used assessment too which represents the gold standard for diagnosis [28,29,30]. It is a tool with strong predictive validity that provides an extremely accurate picture of the current autism spectrum disorder symptoms. In addition, to have an assessment of the developmental framework in early childhood, the Griffiths scale was used to capture the strengths and weaknesses of the areas related to the basis of learning, language, motor skills, and social and emotional aspects [31,32].

### 2.2. Power and Sample Size Calculation

The prevalence of food selectivity in autism ranges from 17% to 83% of children with ASD. This variability of results is attributable, on the one hand, to the different survey methods used by previous studies and, on the other hand, to the lack of a common, unambiguous, and standardized definition of food selectivity.

Assuming a prevalence of food selectivity in the population equal to 17% [11], an incidence in the sample under examination (of ASD children) equal to 28%, considering an alpha significance level of 5%, the minimum number of subjects to enroll in order to have a statistical power of 80%, it turns out to be equal to 103 subjects. This sample, characterized by an adequate level of statistical power, guarantees the representativeness of the population

So, the enrolled sample consists of 111 children, both male and female, with ages between 2 and 10 years and includes 60 children with an autism diagnosis (85% males and 15% females) and 51 children with normotypical development (35% males and 65% females), similar in characteristics, but free of the disorder.

### 2.3. Informed Consent

The parents involved signed informed consent, in accordance with the Declaration of Helsinki; the research protocol was approved by Messina university Ethical committee with Protocol n. 41/23 of 14 February 2023.

All the information on the patient’s medical history, physiological anamnesis and therefore everything concerning the different stages of pregnancy, the pre- and post-natal phase, the acquisition of the first stages of psychomotor development, the rhythm sleep/wakefulness, breastfeeding, weaning and feeding in general, intolerances or allergies, and information on the collection of the remote pathological anamnesis, were recorded.

### 2.4. The Questionnaires

#### 2.4.1. BAMBI Questionnaire

Initially, the completion of a BAMBI questionnaire was requested. This standardized questionnaire consists of 18 items and makes it possible to evaluate behavior during meals, in particular in children with ASD between the ages of 3 and 11, by analyzing three factors: “limited variety”, “refusal of food”, and “characteristics of autism”. This useful and valuable tool, validated for use by different health professionals, examines specific problem behaviors observable in the population of children with autism spectrum disorder and represents a tool of great potential in order to evaluate the different feeding problems in children with ASD. This questionnaire is based on a 1–5 Likert scale, where “1” indicates that the behavior “never” occurs and “5” instead indicates that the behavior “always” occurs during meals. The total score, between 18 and 90, derives from the sum of all the elements; higher scores reflect greater behavioral problems during meals. In the present study, a cutoff score of 34 was used, as suggested by DeMand et al. who studied the psychometric properties of the BAMBI scale in a large representative sample of ASD (ages 2–11 years) [24]. These authors identified four factors: food selectivity, disruptive behavior during meals, refusal of food, and rigidity during meals.

In this questionnaire, some questions refer to disruptive behaviors when food is offered, represented by strong behavioral responses, such as getting up during a meal or throwing food or utensils, or the presence of self-injurious behaviors, typical of autism. Other questions include food selectivity, for which it is asked whether there are behaviors such as turning the head to the other side and closing the mouth on the presentation of unwanted food or a refusal to eat or even taste a variety of foods other than those that they prefer at every meal. The presence of oro-motor difficulties is also evaluated, as children can expel food due to these difficulties. Chewing is the expression of a complex oro-motor skill that, if not properly performed, can contribute to feeding problems. Still within this questionnaire, the presence of sensory processing difficulties is investigated; it is asked if there is a preference for textures, as these preferences are linked to sensory processing deficits. Babies with oral sensory difficulties may have low food registration in the mouth and may expel it.

Although BAMBI can identify the presence of dysphagia, which can occur at different stages of swallowing, the description of it cannot be determined from the parental responses detected by the questionnaire. The same applies to the impact of inadequate oral motor skills, which can only be identified by careful evaluation and advice from healthcare professionals specialized in the field.

#### 2.4.2. Short Sensory Profile Questionnaire

Parents and caregivers of autistic children were also asked to complete an additional questionnaire, the “Short Sensory Profile”, in order to obtain further information related to sensory processing [33,34]. It consists of 38 items, divided into corresponding domains in 7 different areas, which allow us to detect how the ASD child modulates sensory inputs through the sensory systems and which behavioral and emotional responses are associated with sensory processing. Scores are assigned on a five-point Likert scale and range from “always = 0” to “often = 1” to “sometimes = 2” to “rarely = 3” to “never = 4”. Low scores are indicative of frequent dysfunctional behavior. The categories of behaviors that emerge can be classified into “typical performances”, “performances at risk”, or “atypical performances”. The questionnaire was designed as a screening tool and has a discriminating validity of 95% in identifying children with sensory dysfunctions. Children who show difficulty in any of these areas are very likely to have sensory processing problems [35].

#### 2.4.3. ChOMPS Questionnaire

Finally, to analyze the presence of a possible correlation between food selectivity and oro-motor skills, the Child Oral and Motor Proficiency Scale (ChOMPS) was administered, which aims to assess nutrition and related abilities in children between 6 and 7 months of age [36,37,38,39]. The CHOMPS must be completed by a parent or caregiver who is familiar with the child’s typical nutrition and movement skills in relation to the reference age. This investigation tool is also not intended to provide a diagnosis but can instead provide an objective assessment of the child’s feeding and movement abilities in order to facilitate a diagnosis and treatment decision.

### 2.5. Statistical Analysis

Cronbach’s alpha, calculated to measure the internal consistency and reliability of the questionnaire, was found to be equal to 0.892 and therefore have good reliability.

Mean and standard deviation were calculated for each quantitative variable.

The variables considered in the statistical models are the following: age (in year), and the scores deriving from BAMBI, atypical feeding behavior, food selectivity and preferences, oral-motor difficulties, sensory processing difficulties, dysphagia, obsessive eating patterns, requirement of specific food presentation and utensils.

In order to test the normal distribution of numerical variables, the Kolmogorov–Smirnov test was applied. The results revealed the existence of significant differences with respect to the normal condition and, for this reason, the use of non-parametric tests was necessary for a correct and methodologically adequate data analysis.

Preliminarily, Cronbach’s alpha was used to measure the internal consistency and reliability of the questionnaire composed of quantitative questions (items).

In this paper, in order to compare subjects with an ASD diagnosis vs. subjects with a normotypical development, the Non Parametric Combination test [39,40,41] was applied. It is a recent multivariate and multistrata methodology based on permutation solution, that allows to find a correct and consistent estimation of the permutation distributions, both for the partial tests and the combined tests, and to achieve effective solutions of multidimensional hypothesis testing, in the context of non-parametric permutation inference. Once a classification criterion has been established, it checks whether there are statistically significant differences between two or more groups in relation to a set of variables, measured on several statistical units. In this paper the hypotheses system is the following:

$$H_{0}:\;\{{\mathrm{Age}}_{1}\overset{d}{=}{\mathrm{Age}}_{2}\}\cap \ldots \ldots .\cap \{\mathrm{Requir}.\;\mathrm{of}\;\mathrm{specific}\ldots ._{1}\overset{d}{=}\mathrm{Requir}.\;\mathrm{of}\;\mathrm{specific}\ldots {\mathrm{utensiles}}_{2}\;H_{1}:\;\{{\mathrm{Age}}_{1}\overset{d}{\neq }{\mathrm{Age}}_{2}\}\cup \ldots \ldots \cup \{\mathrm{Requir}.\;\mathrm{of}\;\mathrm{specific}\ldots ._{1}\overset{d}{\neq }\mathrm{Requir}.\;\mathrm{of}\;\mathrm{specific}\ldots {\mathrm{utensiles}}_{2}$$

where *d* identifies the “equality” or “inequality” in distribution, 1 identifies the cases group and 2 the controls group, and “Age… Required of specific…utensils” refers to all examined variables measured on two group.

The Spearman coefficient [42] was used to evaluate, for the cases group only, the correlation between the following:

− The total score obtained from the “BAMBI”, the “Short Sensory Profile” and the “ChOMPS” questionnaires.
− Dependent upon the results obtained from the previous correlation analysis, the scores of the single questionnaire items.

Finally, a binary logistic regression model was estimated in order to individuate significant predictors of food selectivity (yes vs. no); the response variable is the dichotomous BAMBI score (food selectivity yes vs. no) and the covariates entered are age, gender, and group (ASD or healthy controls). The results were espressed as Odds Ratio (OR), 95 Confiendece Interval (95 C.I.) and *p*-value.

The statistical package used was NPC test, version 2.0, Statistical Software for Multivariate Nonparametric Permutation Test, Copyright 2001, Methodologica s.r.l. and SPSS for Windows, version 22.0.

A *p*-value < 0.05 was considered statistically significant and reported in bold.

## 3. Results

In Table 1, the mean ± standard deviation and p-values obtained from the NPC test for comparison between cases and controls, in relation to age and to the different scores of BAMBI questionnaire administration, are shown. The only non-significant comparison is referred to as “Requirement of specific food presentation and utensils” (Table 1).

When examining Table 1, is it noted note that the different BAMBI item scores, which are an expression of food selectivity, have significantly higher average score values in the cases than in the controls.

Focusing the attention on cases group, it is calculated mean ± standard deviation of Short Sensory Profile and ChOMPS items scores (Table 2).

Only for the subjects with an ASD diagnosis, Spearman’s Rho correlation coefficient was applied to the total scores obtained from the three administered questionnaires (BAMBI, Short Sensory Profile and ChOMPS) in order to evaluate possible concordance or discordances; Table 3 shows Spearman’s Rho correlation coefficients and *p*-value for correlation between the total scores of abovementioned questionnaires.

The only significance can be observed in the comparison between the total scores of the BAMBI vs. the Short Sensory Profile and, for this reason, the A.A. proceeded by analyzing, by means of the same Spearman coefficient, all the correlations between the scores obtained in the single items of both questionnaires (Table 4).

The obtained results underline a significant link between food selectivity and the items relating to olfactory sensitivity, the sensation-seeking area and the energy area.

Our results fully agree with what is claimed by other authors who have placed the emphasis on the resistance to the consumption of certain types of food derived from the consistency or the smell [43,44]. In fact, sensory alterations are frequent in the autistic population and therefore more correlated with food selectivity.

In this phase, the ChOMPS scores were not considered because no significant correlation was found between ChOMPS and the other questionnaires.

Finally, a binary logistic regression model was estimated in order to individuate significant predictors of food selectivity (yes vs. no), the tested covariates were age, gender and group (ASD or healthy controls). First the univariate models and, later, the multivariable model were estimated to identify the independent factors that were significantly predictive of food selectivity (Table 5).

As can be seen from the results reported in Table 5, the factors that significantly affect the probability of food selectivity are age (a higher age corresponds to a higher probability of being selective), male gender and, above all, ASD. The high value of the OR denotes that ASD subjects have an 86.7 times higher risk of developing food selectivity than children without ASD and this result is highly significant.

## 4. Discussion

This paper initially aimed to describe autism according to the different epidemiological, clinical and diagnostic characteristics, and review the real central focus of the research project, food selectivity. The results obtained from the analyzes support and confirm what is present in the literature which reports that children diagnosed with autism spectrum disorder show difficulty in eating and the presence of food selectivity [45,46,47,48]. Many aspects converge in this concept, which do not refer only to the refusal of food and to a diet with specific preferences and textures, but also to self-harming, aggressive, disruptive behaviors, which manifest themselves with considerable difficulty in remaining seated while eating, little flexibility on meal routines, and obsessive and repetitive patterns [49,50].

Compared to typically developing children, therefore without autistic disorder, the mean values of the scores obtained by autistic children are considerably higher, determining significant differences in almost all domains, except for the preference of certain utensils, or for a particular presentation of food; these results are consistent with what is already present in the literature [51,52,53,54]. Food selectivity can be caused by extreme sensory modulation and sensory problems related to the smell, texture, color and temperature of foods. These sensory processing difficulties have a negative impact on eating behavior, in fact the results obtained underline the correlation between food selectivity and the sensory domain related to taste and smell. The sample included a gender imbalance between the groups that is not related to the study design but is in line with the general epidemiology of the ASD [1,45]. Furthermore, Kelly et al. found that resistance to eating certain foods can be attributed to texture or smell, with excessive olfactory responsiveness making individuals uncomfortable in settings, like school cafeterias, due to the smells of others’ food [21]. Studies comparing oral sensory processing between children with and without autism spectrum disorder, specifically examining the relationship between atypical oral sensory processing, food selectivity, and fruit and vegetable consumption, have shown that children with autism display atypical sensory processing in comparison to their neurotypical peers [55].

Consequently, the meta-analysis conducted underscores the significant differences in sensory processing difficulties experienced by children with autism, even when compared to control groups. Repetitive behaviors and sensory hypersensitivity often manifest together, suggesting a cluster of symptoms related to autism spectrum disorders. However, it is crucial to acknowledge that parents’ behaviors during meals can also influence a child’s eating patterns, as previously discussed in the literature.

Compared to previous studies, there are some advantages of the current research in elucidating potential mechanism of food selectivity in ASD. In fact, the results deriving from statistical analysis highlight a correlation between food selectivity and sensory processing anomalies, in particular of two domains: taste and smell. In addition, another important advantage obtained and a starting point for future investigations, is the correlation between motor skills and the aspect of food selectivity connected to atypical and disruptive behaviors during a meal. A notable strength of this scientific paper is its comprehensive analysis of food selectivity in children with autism spectrum disorder (ASD), drawing upon a wide range of epidemiological, clinical, and diagnostic characteristics. The study effectively consolidates existing literature on the subject, reinforcing the well-documented association between food selectivity and ASD. Additionally, the paper highlights the specific sensory processing domains of taste and smell as crucial factors in food selectivity, shedding light on potential mechanistic links. The inclusion of a meta-analysis further strengthens the findings by revealing significant differences in sensory processing difficulties experienced by children with ASD when compared to control groups. This paper’s emphasis on sensory processing and motor skills in relation to food selectivity opens up new avenues for future research, providing valuable insights that can inform targeted interventions to improve the eating behaviors and overall quality of life for children on the autism spectrum. The main limitation of this scientific paper lies in its focus on observational data and associations without establishing causality. While the study identifies correlations between food selectivity and sensory processing anomalies in children with autism spectrum disorder (ASD), it does not delve into the underlying causal mechanisms. The paper acknowledges the potential influence of parents’ behaviors during meals, but it does not conduct experimental research to pinpoint the precise cause-and-effect relationships between sensory processing, motor skills, and food selectivity in ASD. As a result, the paper provides valuable insights into the characteristics of food selectivity in autistic children but stops short of offering a definitive understanding of the root causes behind these behaviors. Further research is needed to establish causative links and develop targeted interventions.

## 5. Conclusions

Sometimes parents or caregivers may unwittingly be responsible for the child’s continuing dysfunctional eating habits and behaviors. For example, they often take away the food or interrupt the meal by involuntarily teaching the child that when he implements that particular behavior, he will receive an escape from the meal.

In addition to removing unwanted food, the adult can decide to present the child only with his favorite foods, so that he can take in the calories he needs.

In doing so, the child learns that dysfunctional behavior will not only lead to the removal of foods he does not like, but will also receive favorite foods or toys, leading him to maintain his selectivity in the future.

This study provides valuable insights into the complex phenomenon of food selectivity in children diagnosed with autism spectrum disorder (ASD). The research confirms and reinforces existing literature by demonstrating the significant challenges autistic children face in their eating behaviors, encompassing not only food refusal and specific preferences but also encompassing disruptive and self-harming behaviors. Moreover, this paper highlights the pivotal role of sensory processing anomalies, particularly in the domains of taste and smell, in contributing to food selectivity. The meta-analysis strengthens the evidence base by revealing distinct sensory difficulties in children with ASD compared to neurotypical peers. Furthermore, this research suggests a cluster of symptoms related to ASD, including repetitive behaviors and sensory hypersensitivity, and emphasizes the need for a holistic approach to understanding and addressing food selectivity. While acknowledging the influence of parental behaviors during meals, this study paves the way for future investigations to uncover the precise causal mechanisms and develop targeted interventions to improve the mealtime experiences and overall well-being of children on the autism spectrum. In sum, this paper advances our understanding of food selectivity in ASD, providing a foundation for further research and potential therapeutic approaches in this critical area.

Parents may also inadvertently reinforce food refusal through attention. For example, they may ignore the child when he is behaving appropriately, eating quietly, and then pay attention when he engages in food refusal behaviors. It is therefore evident that experts must help parents of children affected by autism and food selectivity problems. The latter can teach specific techniques useful for managing meal times, in order to feed children adequately and improve the approach to the problem, thus lowering the threshold of worry and anxiety.

The tantrums shown by children in the kitchen are a great classic because, for them, it represents a way to claim their freedom through peremptory food choices; when you are a parent of children with autism, everything becomes more difficult.

## Figures and Tables

**Table 1:** Mean ± standard deviation and *p*-values of the comparisons between cases and controls in relation to age and to scores of BAMBY questionnaire.

Variables	Cases	Controls	***p***-Value
Age	5.040 ± 2.496	5.288 ± 2.321	0.134
BAMBI total score	42.100 ± 7.188	25.471 ± 5.039	0.001
Atypical feeding behavior	8.750 ± 3.582	6.235 ± 1.522	0.008
Food selectivity and preferences	22.650 ± 4.637	12.589 ± 3.536	0.001
Oral-motor difficulties	4.050 ± 1.395	2.529 ± 0.943	0.001
Sensory processing difficulties	9.100 ± 2.049	5.823 ± 2.099	0.001
Dysphagia	3.900 ± 1.410	2.529 ± 0.943	0.001
Obsessive eating patterns	3.550 ± 1.731	2.588 ± 0.712	0.032
Requirement of specific food presentation and utensils	1.150 ± 0.671	1.176 ± 0.393	0.883

**Table 2:** Mean ± standard deviation of Short Sensory Profile and ChOMPS items scores.

Questionnaire	Items	Mean ± Standard Deviation
Short Sensory Profile	Tactile sensitivity	27.024 ± 6.221
	Taste/smell sensitivity	10.751 ± 4.845
	Movement sensitivity	13.057 ± 2.568
	Underresponsive/seek sensation	20.307 ± 4.921
	Auditory filtering	20. 056 ± 4.842
	Low energy/weak	27.004 ± 6.375
	Visual/auditory sensitivity	17.401 ± 4.448
	Total score	133.552 ± 22.456
ChOMPS	Complex movement patterns	36.602 ± 8.067
	Basic movement patterns	34.353 ± 9.421
	Oral-motor coordination	24.904 ± 4.559
	Fundamental oral-motor skills	10.403 ± 2.846
	Total score	106.251 ± 21.997

**Table 3:** Spearman’s Rho correlation coefficients and *p*-value for total score.

Comparison	Coefficient	***p***-Value
BAMBI vs. Short Sensory Profile	−0.460	0.041
BAMBI vs. ChOMPS	−0.235	0.318
Short Sensory Profile vs. ChOMPS	0.129	0.587

**Table 4:** Spearman’s Rho correlation coefficients and *p*-value for BAMBI and Short Sensory Profile items scores.

Short Sensory Profile Items									
Tactile				Olfactory	Movement	Seek Sensations	Auditory- Filter	Energy	Auditory- Facial Sensitivity
BAMBI Items	A	Coeff.	−0.314	−0.038	0.099	−0.029	−0.221	0.066	−0.266
		*p*-value	0.177	0.874	0.678	0.903	0.349	0.781	0.258
	B	Coeff.	−0.227	−0.688	−0.194	−0.467	−0.202	0.524	−0.112
		*p*-value	0.336	0.001	0.413	0.038	0.393	0.018	0.639
	C	Coeff.	0.043	0.099	0.389	−0.241	0.065	0.093	0.109
		*p*-value	0.858	0.679	0.090	0.305	0.786	0.696	0.649
	D	Coeff.	0.026	−0.213	0.371	−0.023	0.043	0.110	−0.005
		*p*-value	0.914	0.368	0.107	0.923	0.856	0.645	0.982
	E	Coeff.	0.004	−0.035	0.302	−0.230	−0.015	0.084	0.058
		*p*-value	0.987	0.885	0.198	0.329	0.950	0.724	0.808
	F	Coeff.	−0.213	−0.046	0.038	−0.032	0.378	−0.007	0.226
		*p*-value	0.366	0.846	0.875	0.893	0.101	0.977	0.338
	G	Coeff.	−0.124	0.301	0.062	0.260	0.301	−0.268	−0.020
		*p*-value	0.675	0.198	0.796	0.268	0.199	0.253	0.933

A = Atypical feeding behavior; B = Food selectivity and preferences; C = Oral-motor difficulties; D = Sensory processing difficulties; E = Dysphagia; F = Obsessive eating patterns; G = Requirement of specific food presentation and utensils.

**Table 5:** Univariate and multivariable logistic regression model for food selectivity.

	Univariate Models			Multivariate Model		
Variables	OR	95% C.I.	***p***-Value	OR	95% C.I.	***p***-Value
Age	1.2	1.1–1.4	0.010	1.2	1.0–1.4	0.035
Gender (male)	3.9	1.7–8.9	0.001	3.5	1.5–8.1	0.004
ASD (yes/no)	90.7	23.2–354.9	<0.001	86.7	8.2–920.2	<0.001

## Data Availability

Not applicable.
